# Cyclic-di-AMP signalling in lactic acid bacteria

**DOI:** 10.1093/femsre/fuad025

**Published:** 2023-05-23

**Authors:** Mark S Turner, Yuwei Xiang, Zhao-Xun Liang, Esteban Marcellin, Huong Thi Pham

**Affiliations:** School of Agriculture and Food Sciences, University of Queensland, Brisbane, Queensland 4072, Australia; School of Agriculture and Food Sciences, University of Queensland, Brisbane, Queensland 4072, Australia; School of Biological Sciences, Nanyang Technological University, 639798, Singapore; Australian Institute for Bioengineering and Nanotechnology, University of Queensland, Brisbane, Queensland 4072, Australia; School of Agriculture and Food Sciences, University of Queensland, Brisbane, Queensland 4072, Australia; The University of Danang, University of Science and Technology, Da Nang 50608, Vietnam

**Keywords:** cyclic-di-AMP, lactic acid bacteria, osmotic stress, potassium, compatible solutes

## Abstract

Cyclic dimeric adenosine monophosphate (cyclic-di-AMP) is a nucleotide second messenger present in Gram-positive bacteria, Gram-negative bacteria and some Archaea. The intracellular concentration of cyclic-di-AMP is adjusted in response to environmental and cellular cues, primarily through the activities of synthesis and degradation enzymes. It performs its role by binding to protein and riboswitch receptors, many of which contribute to osmoregulation. Imbalances in cyclic-di-AMP can lead to pleiotropic phenotypes, affecting aspects such as growth, biofilm formation, virulence, and resistance to osmotic, acid, and antibiotic stressors. This review focuses on cyclic-di-AMP signalling in lactic acid bacteria (LAB) incorporating recent experimental discoveries and presenting a genomic analysis of signalling components from a variety of LAB, including those found in food, and commensal, probiotic, and pathogenic species. All LAB possess enzymes for the synthesis and degradation of cyclic-di-AMP, but are highly variable with regards to the receptors they possess. Studies in *Lactococcus* and *Streptococcus* have revealed a conserved function for cyclic-di-AMP in inhibiting the transport of potassium and glycine betaine, either through direct binding to transporters or to a transcriptional regulator. Structural analysis of several cyclic-di-AMP receptors from LAB has also provided insights into how this nucleotide exerts its influence.

## Teaser

This review examines components of the cyclic dimeric adenosine monophosphate signalling system in lactic acid bacteria and explores the roles this second messenger plays in growth and stress resistance.

## Introduction

Lactic acid bacteria (LAB) include a diverse range of bacteria commonly found in the environment on vegetal and animal sources and in raw/fresh foods. Generally regarded as safe LAB play important roles in food fermentations assisting with the preservation and transformation of milk, vegetable, meat, legume, and cereal substrates. The desire to better control fermentations has driven the selection of starter and adjunct cultures with optimal acidification, flavour development, and texture alteration. Certain LAB are also major contributors to food spoilage, particularly under storage conditions with reduced oxygen such as modified atmosphere or vacuum packaging. LAB form an important component of the commensal microbial population on various mucosal surfaces of humans and animals, including the oral cavity, gastrointestinal, and urogenital tracts. In addition, a number of LAB species are major pathogens causing significant morbidity and mortality.

LAB are commonly exposed to external challenges in their ecological niches, during food processing, following consumption or during infection, which may limit their growth or survival. This may include acid stress during food fermentation or transit through the stomach, osmotic stress following salt addition in cheese-making, heat stress during spray drying, nutrient stress encountered towards the end of a food fermentation, or immune system attack during infection (van de Guchte et al. 2002, Tsakalidou and Papadimitriou 2011). Like all bacteria, LAB have systems that allow them to sense external stimuli and adapt as best as possible. These can result in changes at the transcriptional (RNA level) and/or post-transcriptional (protein level or activity) levels, which result in downstream physiological or structural changes in the cell.

Second messenger signalling systems in bacteria involve sensing of an external (first) signal, which causes changes in the activity of a synthesis or degradation enzyme that modulates the concentration of an intracellular (second) signal molecule (Yoon and Waters 2021). The best characterized second messengers include nucleotides cyclic adenosine monophosphate (cAMP) and guanine tetra- or penta-phosphate [(p)ppGpp] that are involved in carbon metabolism and the stringent response, respectively. More recently, cyclic dinucleotide monophosphate second messengers have been characterized.

**Figure 1.**
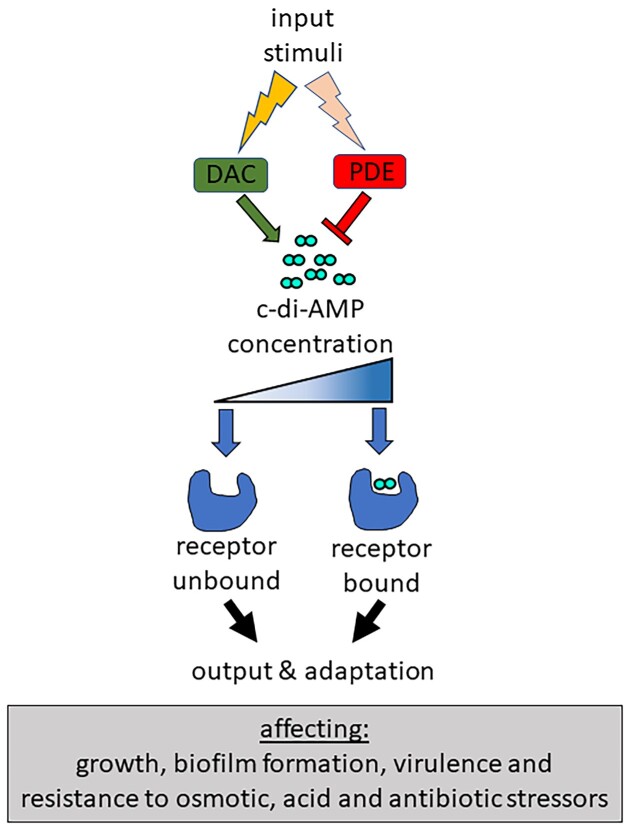
Overview of the c-di-AMP signalling system. The c-di-AMP synthesis and degradation enzymes respond to external or cellular stimuli resulting in changes in c-di-AMP levels. Upon reaching a certain intracellular concentration, c-di-AMP binds to receptors leading to activation or inhibition of activity and a subsequent cellular output and adaptation.

These include cyclic dimeric guanosine monophosphate (c-di-GMP), cyclic-di-AMP (c-di-AMP), and cyclic GMP–AMP (cGAMP), and very recently cyclic trinucleotide monophosphate signalling molecules have also been identified (Yoon and Waters 2021). C-di-GMP plays an important role in the motile/sessile lifestyle transition, cell shape, and virulence (Jenal et al. 2017). Second messengers cGAMP and other cyclic mono-/di-/tri-nucleotides trigger altruistic cell suicide following bacteriophage infection, thereby preventing viral replication (Duncan-Lowey and Kranzusch 2022). LAB produce c-di-AMP, but have not been found to synthesize other cyclic di- or tri-nucleotides. C-di-AMP was first discovered in 2008 (Witte et al. 2008) and since then there has been a significant amount of new insight into how it exerts its control in Gram-positive bacteria, Gram-negative bacteria and Archaea. This includes how input signals feed into the system, the receptors that c-di-AMP binds and the resultant cellular outputs/phenotypes (Fig. 1). This review will focus on recent learnings of c-di-AMP signalling systems specifically from work done in LAB species that are of importance in food applications, as colonizers of mucosal surfaces or causes of disease. It will include a bioinformatic analysis of c-di-AMP signalling components in representative LAB genomes, the physiological changes that occur upon c-di-AMP concentration shifts and stimuli that trigger c-di-AMP level changes. For a broader understanding of c-di-AMP signalling systems outside of LAB, the reader is referred to several recent reviews (Commichau et al. 2019, He et al. 2020, Stülke and Krüger 2020, Yin et al. 2020, Zarrella and Bai 2020).

## C-di-AMP and pool size regulation

### C-di-AMP synthesis by the conditionally essential CdaA

**Figure 2.**
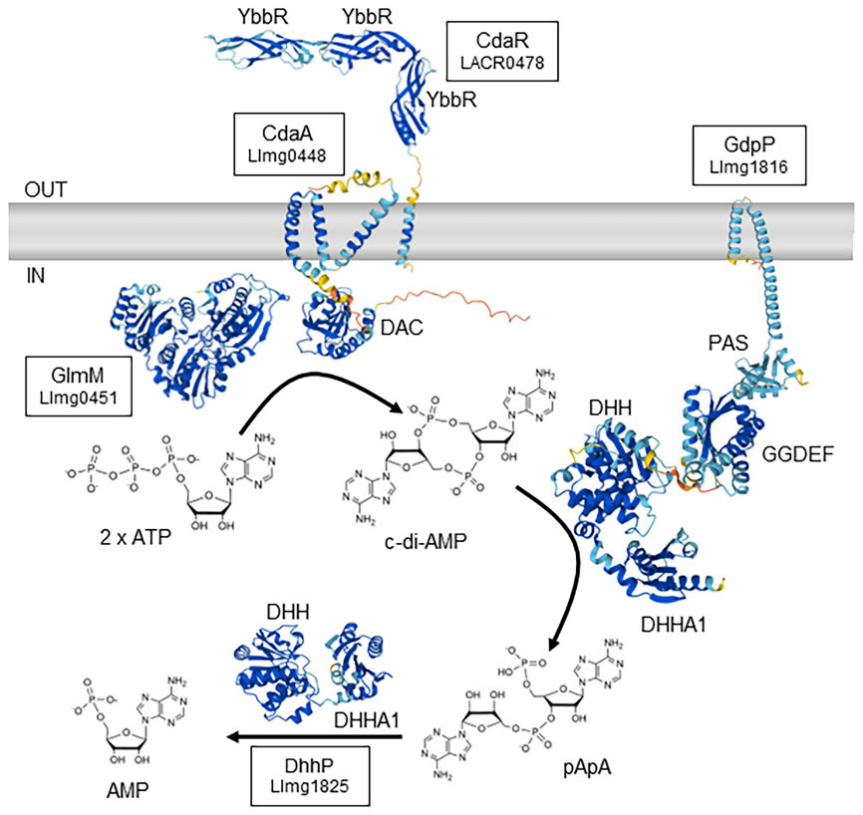
Synthesis and degradation of c-di-AMP with predicted structures of proteins. Uniprot accessions are from *L. cremoris* strains: A2RIF7 (CdaA; Llmg_0448), Q031P3 (CdaR; LACR_0478), A2RM60 (GdpP; Llmg_1816), A2RIG0 (GlmM; Llmg_0451), and A2RM69 (DhhP; Llmg_1825). AlphaFold predictions of protein tertiary structures and Rhea chemical structures are provided under a Creative Commons Attribution (CC BY 4.0) License (Jumper et al. 2021, Bansal et al. 2022, Varadi et al. 2022). Colour coding in protein structures indicates the confidence of the prediction by AlphaFold (dark blue = very high confidence; light blue = confident; yellow = low confidence; orange = very low confidence). Note that only monomers are shown for simplicity.

**Figure 3.**
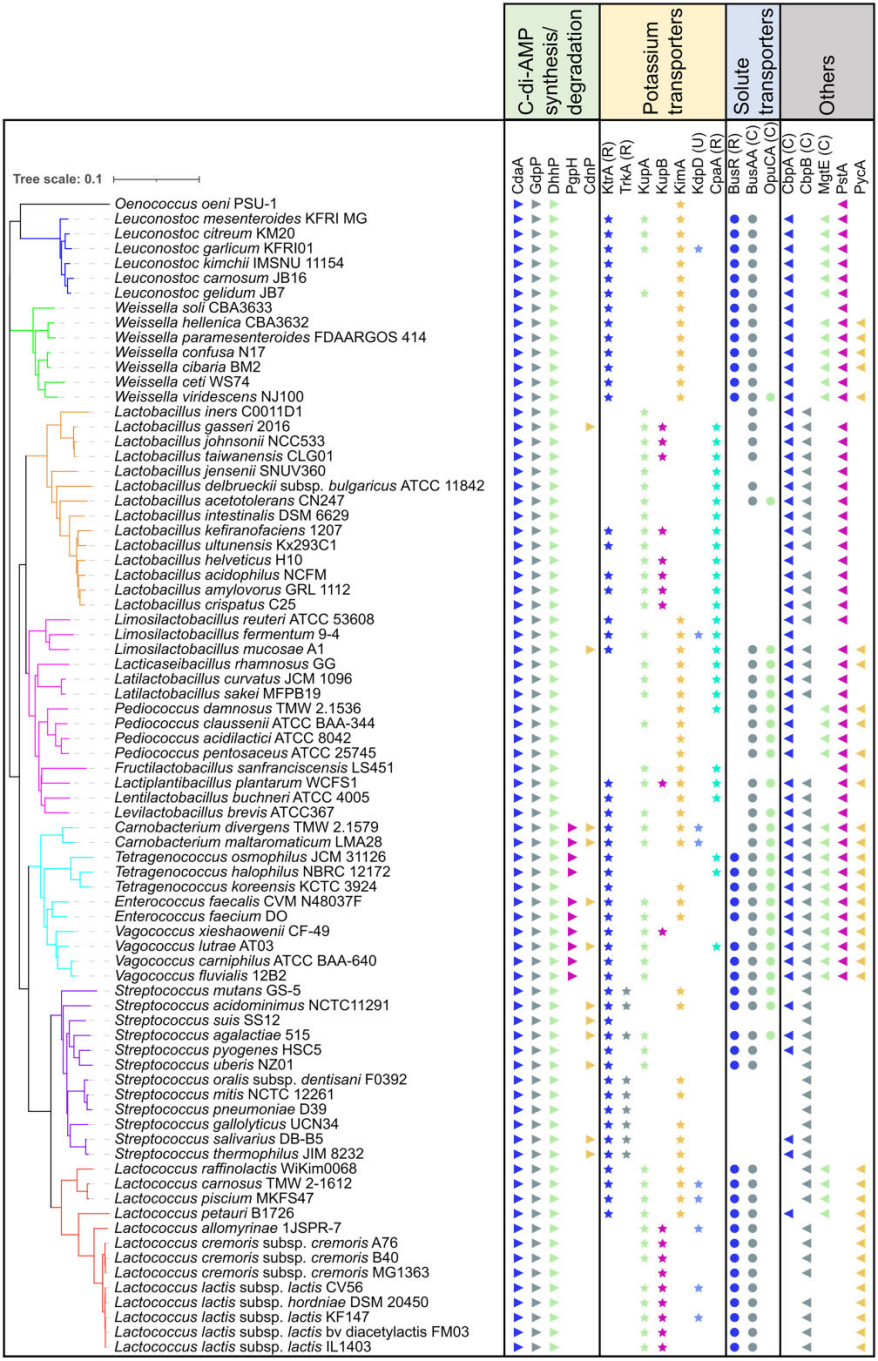
Presence and analysis of c-di-AMP signalling components in representative LAB. Representative taxa genomes of LAB were selected from the NCBI database. A phylogenetic tree was constructed using FastME 2.1.6.1 (Lefort et al. 2015), which was based on the Genome BLAST Distance Phylogeny (GBDP) distances. These distances were calculated from 16S rRNA gene sequences utilizing the TYGS platform (Meier-Kolthoff and Goker 2019). The iTOL software (Letunic and Bork 2021) was used to visualize the phylogenetic tree and receptors. For each strain, we searched for homologs of c-di-AMP synthesis and degradation enzymes, and their receptors using the custom BLAST function in Geneious Prime software version 2022.1.1 (Biomatters Ltd, New Zealand). This search was compared with published c-di-AMP receptors that originated from either LAB or other c-di-AMP-producing bacteria. Specific c-di-AMP binding domains are denoted as follows: RCK_C domain (R), CBS domain (C), and USP domain (U).

The intracellular level of c-di-AMP is regulated by enzymes involved in its synthesis and degradation as well as active export from the cell. LAB, like most *Firmicutes*, have only one diadenylate cyclase (DAC) enzyme (IPR034701) called CdaA (or DacA) that synthesizes one c-di-AMP from two molecules of ATP (Figs. 2 and 3). This contrasts to the more complicated c-di-GMP signalling system where bacteria can contain dozens of synthesis and degradation enzymes (Sondermann et al. 2012). CdaA contains an N-terminal domain consisting of three predicted transmembrane spanning regions and a C-terminal cytoplasmic enzymatic domain with Asp–Gly–Ala (DGA) and Arg–His–Arg (RHR) motifs in the active site. Structural analysis suggests that CdaA dimers likely need to form oligomeric complexes in order to form a catalytically active enzyme and prevention of oligomer formation is one way to inhibit c-di-AMP synthesis (Pathania et al. 2021). Levels of intracellular c-di-AMP in *Bacillus subtilis* have been estimated to be ∼2 µM (Oppenheimer-Shaanan et al. 2011).

The first demonstration of c-di-AMP production in a LAB was reported in 2011 (Kamegaya et al. 2011). C-di-AMP was identified in cell extracts of *Streptococcus pyogenes* and reaction products from purified *S. pyogenes* CdaA using high-pressure liquid chromatography fractionation and matrix-assisted laser desorption ionization–time of flight mass spectrum analysis. This work also reported that attempts to inactivate the *cdaA* gene were unsuccessful. Several other studies have since reported the essentiality of *cdaA* in LAB (Song et al. 2005, Bai et al. 2013, Devaux et al. 2018, Kundra et al. 2021) and other bacteria (Whiteley et al. 2015, Gundlach et al. 2017, Zeden et al. 2018) under normal laboratory growth conditions, suggesting that cells devoid of c-di-AMP are unable to survive or multiply.

Like in other Firmicutes, the c-di-AMP synthesis gene *cdaA* is encoded in a highly conserved three-gene operon in LAB (Pham et al. 2016). Downstream of *cdaA* is *cdaR*, which encodes a protein of unknown function that contains a transmembrane domain (TMD) and three or four YbbR domains (IPR012505) located on the external side of the cytoplasmic membrane. CdaR directly interacts with CdaA through interactions between their TMDs (Rismondo et al. 2016, Gibhardt et al. 2020; Fig. 2). Interestingly, in some streptococcal strains, the CdaA and CdaR proteins are fused together (e.g. A5LQF8, E1M7C3, and V8IIN0). AlphaFold predictions of these large fusion proteins in UniProt indicate that the first TMD of CdaA may be the interacting domain with the TMD of CdaR. The function of CdaR is not known at present, but it appears to play a role in regulating c-di-AMP synthesis by CdaA.

GlmM is an essential phosphoglucosamine mutase that interconverts glucosamine-6-phosphate and glucosamine-1-phosphate, forming an early step in the synthesis of cell-wall peptidoglycan and other cell-wall polymers (Barreteau et al. 2008). In *Lactococcus lactis*, GlmM binds directly to CdaA and inhibits c-di-AMP synthesis activity (Zhu et al. 2016; Fig. 2). This inhibitory activity of GlmM is shared broadly in Firmicutes and recent structural analysis has indicated that GlmM binding prevents formation of catalytically active CdaA oligomers (Tosi et al. 2019, Gibhardt et al. 2020, Pathania et al. 2021). In suppressor mutant screening studies, a single amino acid change (I154F) in *L. lactis* GlmM resulted in enhanced binding to CdaA and greater inhibition of activity (Zhu et al. 2016). Structural analysis of CdaA–GlmM complexes in other Firmicutes identified this residue is in close proximity to the contact site between the two proteins (Tosi et al. 2019, Pathania et al. 2021). The functional significance of the CdaA–GlmM interaction, however, remains unclear, but does suggest that there is likely coordination between c-di-AMP and peptidoglycan precursor synthesis. This hypothesis is supported by the finding of elevated intracellular levels of the peptidoglycan precursor UDP-*N*-acetylglucosamine in *L. lactis* mutants with high c-di-AMP (Zhu et al. 2016, Pham et al. 2021).

### C-di-AMP hydrolysis

Degradation of c-di-AMP to phosphoadenylyl adenosine (5′-pApA) and to AMP occurs through the actions of phosphodiesterases (PDEs). In LAB, the most common PDEs are the GGDEF domain protein containing phosphodiesterase (GdpP, formerly YybT; IPR014528) and DHH–DHHA1 domain protein (DhhP; IPR038763) PDEs. These both contain a DHH domain, that contains highly conserved active site Asp–His–His residues, and a DHH-associated (DHHA1) domain at the C-terminus (Rao et al. 2010, Corrigan et al. 2011, Ye et al. 2014, Huynh and Woodward 2016). GdpP is tethered to the membrane via two N-terminal TMDs and contains two potential regulatory domains in the central part of the protein. These include a heme-binding Per-ARNT-Sim (PAS) domain and a region structurally similar to GGDEF domains, which has weak ATPase activity (Rao et al. 2011, Tan et al. 2013; Fig. 2). DhhP contains only a DHH–DHHA1 domain and lacks any obvious transmembrane or regulatory domains. GdpP is a highly selective cyclic-dinucleotide hydrolase with high affinity for c-di-AMP (Rao et al. 2010, Huynh and Woodward 2016). DhhP, on the other hand, has promiscuous substrate specificity cleaving both c-di-AMP or 5′-pApA into AMP *in vitro* (Huynh and Woodward 2016). In some cases, such as for *Streptococcus pneumoniae* DhhP, it can also cleave c-di-AMP directly to AMP *in vitro*, but with 1000-fold lower activity compared with its conversion of pApA to AMP (Bai et al. 2013). In several LAB, including *S. pneumoniae, Streptococcus mitis*, and *Enterococcus faecalis*, inactivation of both *gdpP* and *dhhP* genes result in significantly greater c-di-AMP levels compared to mutants with only one of these genes inactivated (Bai et al. 2013, Rørvik et al. 2020, Kundra et al. 2021). Interestingly, under biofilm-like growth conditions with 5% CO_2_, a *S. pneumoniae dhhP* mutant, but not a *gdpP* mutant, contained a very high c-di-AMP level (Wooten et al. 2020). In other LAB, however, inactivation of just *gdpP* results in very high (20–30-fold) c-di-AMP levels compared with the wild-type (Pham et al. 2018, 2021). It is possible that in some bacteria, pApA may act as a feedback inhibitor of PDE activity, which results in elevated c-di-AMP upon loss of DhhP activity. Stand-alone DHH–DHHA1 homologs are commonly present in organisms that do not produce c-di-AMP, so their role specifically in c-di-AMP hydrolysis *in vivo* requires further investigation (Huynh and Woodward 2016).

A few LAB genera, including *Enterocococus, Carnobacterium, Tetragenococcus*, and *Vagococcus* contain an additional c-di-AMP PDE called PgpH (PF07698; Fig. 3). This protein consists of a C-terminal intracellular HD domain (IPR006674) with a His–Asp motif in the active site that degrades c-di-AMP (Huynh et al. 2015). PgpH contains seven transmembrane spanning regions (IPR011621) in the central part and a large extracellular N-terminal domain (IPR011624) of unknown function. There have been no experimental studies of PgpH homologs in LAB to date.

Like for DACs, there are several regulators of PDE enzyme activity that allow the cell to modulate c-di-AMP levels in response to intracellular and extracellular stimuli. The stringent response regulator (p)ppGpp strongly inhibits c-di-AMP hydrolysis by GdpP and PgpH in several Firmicutes (Rao et al. 2010, Huynh et al. 2015). Limited biochemical studies on LAB GdpP regulation have shown that (p)ppGpp is a very weak inhibitor of GdpP from *E. faecalis in vitro* (Wang et al. 2017). GdpP contains a PAS domain, which has been shown to bind heme (Rao et al. 2011, Tan et al. 2013), and this widespread sensory domain has been found to sense redox potential, light, metal ions, and oxygen in other proteins (Stuffle et al. 2021). The PDE activity of the DHH–DHHA1 domain only from *E. faecalis* GdpP was around 13-fold higher than the full-length protein suggesting a role for the PAS and/or GGDEF domains in dampening c-di-AMP hydrolysis (Wang et al. 2017). However, truncation of the PAS domain in the *S. pneumoniae* GdpP did not affect PDE activity (Bai et al. 2013). The degenerate GGDEF domain from *E. faecalis* GdpP has been found to exhibit weak ATPase activity (Wang et al. 2017), however, if and how this affects c-di-AMP hydrolysis is not clear. In a screen for heat-resistant mutants in *L. lactis*, an A285D mutation was identified in *gdpP*, which is located in the GGDEF domain, suggesting this part of the protein can influence c-di-AMP degrading activity of the DHH–DHHA1 domain (Smith et al. 2012). The degenerate GGDEF domain shares structural similarity with diguanylate cyclase enzymes, including DgcR and WspR (De et al. 2009, Teixeira et al. 2021), providing further support for a nucleotide-binding function. Further work is required to determine what roles the PAS and degenerate GGDEF domains in GdpP and the extracellular domain in PgpH play in sensing signals and adjusting PDE activity.

### C-di-AMP export

In addition to enzymatic degradation of c-di-AMP by PDEs, the intracellular level of this signalling molecule can also be lowered physically, by active export from the cell. Multidrug resistance (MDRs) transporters of the major facilitator superfamily (IPR011701), which are widespread in LAB have been shown to secrete c-di-AMP (Woodward et al. 2010, Huynh and Woodward 2016). Work in *Listeria* and *Bacillus* has revealed that mutants with increased expression of MDRs or inactivated MDR genes secreted higher or lower levels of c-di-AMP, respectively, as determined by direct measurement or indirectly by measuring beta interferon (IFN-β) induction in infected macrophages (Crimmins et al. 2008, Woodward et al. 2010, Schwartz et al. 2012, Yamamoto et al. 2012, Kaplan Zeevi et al. 2013, Townsley et al. 2018). In *L. lactis*, mutations that increased transcription of an EmrB-like MDR gene (*llmg_1210*) by several hundred-fold resulted in reduced intracellular c-di-AMP levels and proportionally higher extracellular c-di-AMP levels (Pham et al. 2018). This lowering of the intracellular c-di-AMP level was significant enough to bring about a phenotypic change in salt resistance in *L. lactis*. It is, however, currently not known if more subtle (non-mutational) changes in MDR expression or activity can occur in bacteria in response to environmental changes, which lead to c-di-AMP level fluctuations that are significant enough to alter either bacterial physiology or host immune responses.

## Phenotypes of LAB mutants with low or high c-di-AMP levels

A plethora of phenotypic changes in LAB mutants with altered c-di-AMP levels have been reported (Supplementary Table S1). Mutants with high c-di-AMP have been obtained by inactivation of the *gdpP* alone or both *gdpP* and *dhhP*. Despite its essentiality in some bacteria under normal growth conditions, targeted inactivation of *cdaA* in wild-type LAB backgrounds or by selection of suppressor mutants from high c-di-AMP mutant strain backgrounds have allowed for the study of cells either devoid of c-di-AMP, or partially defective in its synthesis.

Some contrasting phenotypic results for mutants with high or low/no c-di-AMP have been reported in different LAB. This is perhaps due in part to methodological differences, such as growth versus killing assays or different media (osmolarity or solute composition) being used. It should also be mentioned here that mutants with very high or low c-di-AMP can be unstable and can easily accumulate suppressor mutations (Gundlach et al. 2015, Whiteley et al. 2015, Whiteley et al. 2017). It is not known what the ‘normal’ range of c-di-AMP concentration is within cells whereby suppressor mutations will not occur, but this likely fluctuates depending upon the growth conditions and specific bacterial species. In our lab, prolonged incubation of a high c-di-AMP *gdpP* mutant of *L. lactis* under normal (non-stressed) growth conditions during plasmid transformation and excision experiments commonly resulted in the accumulation of suppressor mutations (Choi et al. 2017). We therefore advise caution when working with mutants with altered c-di-AMP levels. This can be done by checking the c-di-AMP concentration, monitoring phenotypes, and carrying out whole genome sequencing.

### Growth and osmoregulation

A commonly observed phenotypic change for LAB mutants with no/low c-di-AMP and also high c-di-AMP is growth impairment (Supplementary Table S1). This indicates that a tuned c-di-AMP level is necessary for optimal cell multiplication under specific environmental conditions. As such, c-di-AMP has been referred to as an ‘essential poison’ (Gundlach et al. 2015). Studies in *Streptococcus agalactiae* and *E. faecalis* identified that *cdaA*, and therefore c-di-AMP, becomes dispensable for growth during anaerobic culturing or on media devoid of osmoprotectants, namely, glycine betaine or carnitine (Devaux et al. 2018, Kundra et al. 2021). In agreement with the former condition, inactivation of *cdaA* was successful in several *Streptococcus* species (*mutans, sanguinis, mitis*, and *pyogenes*) under anaerobic or microaerophilic culturing conditions (Xu et al. 2011, Cheng et al. 2016, Fahmi et al. 2019, Rørvik et al. 2020). A thorough understanding of why O_2_ is toxic to *cdaA* mutants of LAB is not yet apparent, but it has also been reported in other Firmicutes, including *Staphylococcus aureus* (Zeden et al. 2018). The toxicity of osmoprotectants to *cdaA* mutants appears to be due to their uncontrolled accumulation in the absence of c-di-AMP control, which triggers osmotic instability. In several LAB, growth of *cdaA* mutants in rich growth media can be improved by the addition of elevated salt concentrations (Zhu et al. 2016, Kundra et al. 2021). Conversely, elevated salt addition to growth media results in poor growth of high c-di-AMP LAB mutants (Smith et al. 2012, Devaux et al. 2018, Zarrella et al. 2018, Teh et al. 2019). The role of c-di-AMP in osmoregulation has been well established in numerous bacteria, which can be explained by a number of c-di-AMP receptors being involved in potassium and compatible solute transport.

A likely consequence of dysregulated osmotic pressure is changes in phenotypes affected by turgor pressure such as cell lysis, cell size, and cell chain length. CdaA mutants in *L. lactis* and *E. faecalis* have been found to be more prone to cell lysis during normal growth (Kundra et al. 2021, Pham et al. 2021). In several LAB, the size of cells was negatively influenced by c-di-AMP, with high c-di-AMP mutants having smaller cells (Teh et al. 2019) and low/no c-di-AMP cells have enlarged cells (Kundra et al. 2021). This agrees with lysis and cell size changes observed in other Firmicutes (Corrigan et al. 2011, Gundlach et al. 2017, Zeden et al. 2018), including the striking cell elongation differences recently reported for DAC and PDE mutants of *Clostridioides difficile* (Oberkampf et al. 2022). Abnormal changes in cell size may be connected with growth defects of mutants with altered c-di-AMP levels.

### Biofilm formation, colonization, and virulence

A number of studies have identified effects of the level of c-di-AMP in LAB and phenotypes associated with pathogenicity, including biofilm formation, colonization, and virulence in various model systems (Supplementary Table S1). In several studies, both high or low/no c-di-AMP levels have been shown to result in phenotypes that would be predicted to reduce pathogenicity of LAB. Despite this, there is, however, a lack of understanding of the mechanisms by which c-di-AMP impacts colonization and pathogenicity. The role of c-di-AMP in biofilm formation of LAB is variable between LAB and even between studies of the same species (Zarrella and Bai 2020). The mechanistic basis for how c-di-AMP affects biofilm formation in *S. mutans* has been investigated the most. A high c-di-AMP mutant of *S. mutans* forms greater biofilm biomass and produces more exopolysaccharide (EPS) by elevating expression of the glucosyltransferase GtfB (Peng et al. 2016). It was found that the TrkA_C domain containing c-di-AMP binding protein CabPA (a TrkA homolog) binds to the response regulator VicR, which regulates *gtfB* transcription (Peng et al. 2016). Biofilm work in another study in *S. mutans* reported conflicting results, with a *cdaA* mutant found to produce greater biofilm biomass, EPS production and GtfB expression (Cheng et al. 2016). Furthermore, a study in *S. mutans* found no difference in biofilm formation of a *gdpP* mutant (Konno et al. 2018). It has been suggested that strain differences and/or culture conditions may account for these variable results (Zarrella and Bai 2020). In fermented foods, EPS can enhance the viscosity and mouthfeel, therefore it would be of interest to determine if c-di-AMP exerts any influence over EPS enzyme expression in industrial LAB.

In *S. pyogenes*, a *cdaA* mutant is unable to secrete the secreted cysteine protease virulence factor SpeB (Fahmi et al. 2019). Interestingly, a suppressor mutation resulting in inactivation of the K^+^ transporter KtrB restored SpeB production (Faozia et al. 2021). KtrB forms a complex with, and is regulated by the c-di-AMP binding RCK_C gating component KtrA (discussed below). SpeB production changes were found to be due to K^+^ imbalance since the addition of sub-growth inhibitory concentrations of ionophores valinomycin and gramicidin restored production in a *cdaA* mutant (Faozia et al. 2021). A number of other phenotypes (e.g. biofilm formation, acid resistance, and ampicillin resistance) of the *S. pyogenes cdaA* mutant were either partially or fully restored to wild-type levels upon inactivation of KtrB, demonstrating the significant negative impact of uncontrolled high intracellular K^+^ levels in cells devoid of c-di-AMP (Faozia et al. 2021). However, the virulence of the *cdaA*/*ktrB* mutant in a subcutaneous mouse model remained similarly attenuated as the *cdaA* mutant, indicating that other c-di-AMP controlled physiological or structural changes beyond K^+^ balance influence infectivity of *S. pyogenes*.

Due to its importance in cell growth and virulence, there has been interest in screening of small molecules, which inhibit DAC activity in pathogens (Opoku-Temeng et al. 2016). A DAC inhibitor compound called ST056083, which was identified from a 1000 compound library (Zheng et al. 2014), was shown to inhibit c-di-AMP levels, EPS production, and biofilm formation in *E. faecalis* (Chen et al. 2018). In other work, an inhibitor called IPA-3 was identified from a screen of 1133 compounds using the *Streptococcus suis* CdaA enzyme (Li et al. 2022). This compound was able to inhibit the growth of a number of different c-di-AMP producing Gram-positive bacteria, but not *E. coli*, which does not make c-di-AMP (Li et al. 2022).

### Antibiotic resistance

Several studies have reported changes in resistance towards antimicrobials in LAB due to an imbalance of c-di-AMP. This includes mostly cell envelope targeting antimicrobials such as ampicillin, cefuroxime, vancomycin, bacitracin, daptomycin, and cell-wall hydrolytic enzymes (Smith et al. 2012, Fahmi et al. 2019, Kundra et al. 2021, Pham et al. 2021, Rørvik et al. 2021). However, in most cases, the mechanisms by which c-di-AMP affects antimicrobial resistance is unclear.

Recent work in *L. lactis* has explored the mechanisms underlying the sensitivity of a low c-di-AMP *cdaA* mutant towards the β-lactam antibiotic cefuroxime (Pham et al. 2021). Using a genetic suppressor screen, mutations that either partially or fully inactivated the K^+^ importer KupB or the glutamine importer GlnPQ restored cefuroxime resistance. The control of KupB by c-di-AMP (discussed below) and the importance of K^+^ in osmoregulation in *L. lactis* had been demonstrated before (Pham et al. 2018, Quintana et al. 2019). The connection between c-di-AMP and GlnPQ, however, was new. It was found that c-di-AMP inhibits GlnPQ activity indirectly by its control of K^+^ uptake. Elevated ionic strength activation on GlnPQ activity was identified earlier by *in vitro* experiments using reconstituted proteoliposomes (Schuurman-Wolters and Poolman 2005). Imported glutamine is rapidly converted to glutamate that can be converted to aspartate. The anionic amino acids aspartate and glutamate are the most abundant free amino acids in *L. lactis* (Pham et al. 2021). Cefuroxime resistance was found to be inversely related with osmoresistance. Together, this work showed that uncontrolled accumulation of turgor-inducing intracellular osmolytes (K^+^ or anionic amino acids) causes cefuroxime sensitivity in low c-di-AMP cells. This appears to be an example of where dysregulation of osmotic homeostasis indirectly results in another phenotypic difference.

## C-di-AMP receptors in LAB

**Figure 4.**
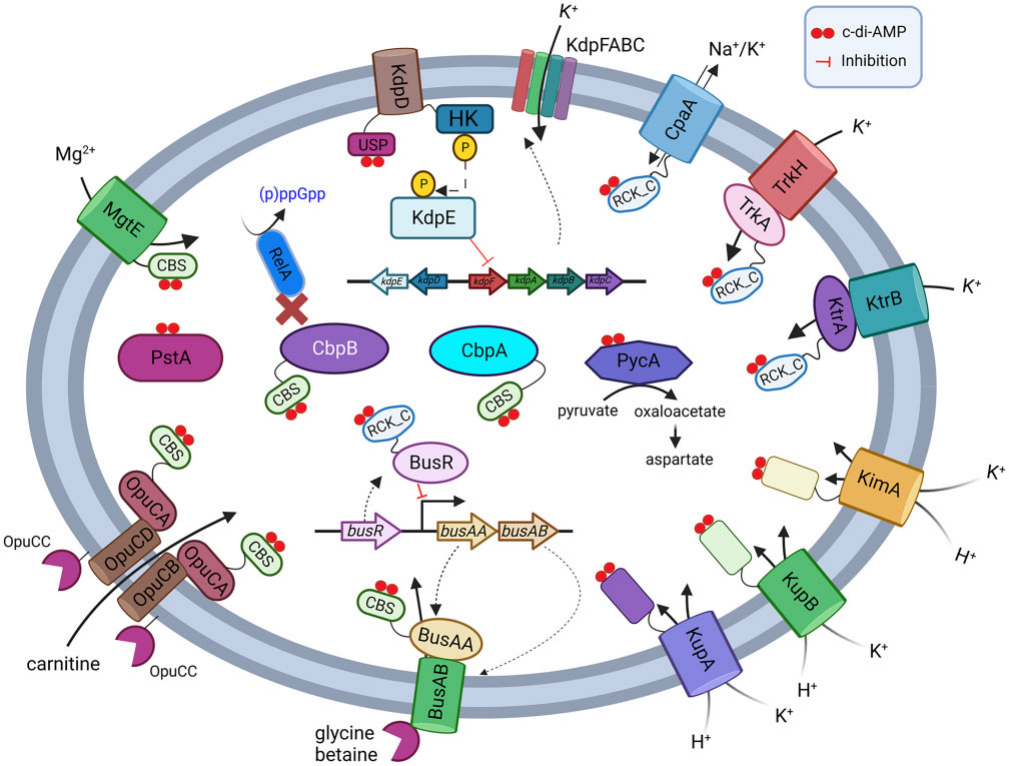
Overview of c-di-AMP receptors found in LAB with binding domains and functions indicated. Created with BioRender.com.

C-di-AMP exerts its control through the binding of protein and riboswitch receptors (He et al. 2020, Stülke and Krüger 2020; Figs. 1 and 4). A number of protein receptors from LAB have been characterized, but LAB do not appear to possess the c-di-AMP binding riboswitch (Nelson et al. 2013). Methods to find c-di-AMP binding partners have included screening of protein expression libraries (Corrigan et al. 2013, Schuster et al. 2016) and affinity purification using c-di-AMP coupled beads or resin (Bai et al. 2014, Sureka et al. 2014, Peng et al. 2016). Suppressor mutant screening approaches using high or low c-di-AMP mutants have also identified genes that encode c-di-AMP receptors (Devaux et al. 2018, Pham et al. 2018). Following the identification of c-di-AMP binding domains, targeted testing of proteins from different bacteria has been carried out. The most commonly used method of verifying a c-di-AMP receptor involves the differential radial capillary action of ligand assay (DRaCALA; Roelofs et al. 2011). This involves mixing ^32^P-radiolabelled c-di-AMP with the protein, then spotting the mixture on a nitrocellulose membrane. The protein will bind to the membrane at the site of application and c-di-AMP will diffuse beyond this site, unless it is bound by the protein. This method can be used to quantitatively determine the dissociation constant (K_d_) and can also test binding of unpurified receptors from *E. coli* whole cell lysates (Roelofs et al. 2011). Several different c-di-AMP binding domains have been identified. These include the cystathionine-β-synthase (CBS, [IPR000644]), regulator of K^+^ conductance (RCK_C; [IPR006037]), and universal stress protein (USP; [IPR014729]) domains, which are present within proteins that play roles in osmoregulation (He et al. 2020, Stülke and Krüger 2020; Fig. 4). C-di-AMP binding pockets have also been identified in proteins without designated binding domains, which allow for allosteric inhibition of enzyme activity (Sureka et al. 2014).

### Potassium transporters

Potassium is the dominant cation in bacteria, present at intracellular concentrations of several hundred millimolars, playing a critical role in osmoregulation, pH control, protein expression, and enzyme activity (Stautz et al. 2021). A wide variety of different c-di-AMP receptors identified in bacteria are involved in potassium homeostasis (Stülke and Krüger 2020). These include K^+^ importers or importer gating components (TrkA, TrkH, KupA, KupB, and KimA), two-component systems that regulate K^+^ importer expression (KdpD), a K^+^ exporter (CpaA), and a riboswitch that regulates K^+^ importer gene transcription. Some of these K^+^ transporters contain distinct c-di-AMP binding domains, including RCK_C and USP. In LAB, various combinations of different c-di-AMP receptors involved in K^+^ homeostasis are present, with some species containing five (*Lactiplantibacillus plantarum* WCFS1 and *Limosilactobacillus fermentum* 9–4) and others containing just one (*Oenococcus oeni* PSU1, *Lactobacillus iners* C0011D1, *Pediococcus acidilactici* ATCC8042, *Pediococcus pentosaceus* ATCC25745, and *L. lactis* MG1363; Fig. 3). There is also variation between strains of the same species, with some strains of *L. lactis* containing three putative K^+^ transporters regulated by c-di-AMP (Pham and Turner 2019).

The first demonstration of a c-di-AMP receptor in LAB was the RCK_C domain containing CabP (KtrA) from *S. pneumoniae*, which was identified by passing cell lysates through a c-di-AMP agarose resin (Bai et al. 2014). KtrA and TrkA homologs are termed gating components since they bind to and regulate the activity of membrane imbedded K^+^ translocation subunits KtrB and TrkH, respectively (Stautz et al. 2021). KtrA contains one RCK_C domain and forms an octameric ring, whilst TrkA contains two RCK_C domains and forms a tetrameric ring (Stautz et al. 2021). Work in *S. pneumoniae* demonstrated that the interaction between CabP and KtrB and K^+^ uptake from this import system was reduced in the presence of c-di-AMP (Bai et al. 2014). Subsequent investigations have confirmed KtrA and TrkA homologs bind c-di-AMP in *S. mutans* and *S. agalactiae* (Peng et al. 2016, Devaux et al. 2018). KtrA and TrkA homologs in *S. mutans* had K_d_ values of 7.8 and 1.2 µM, respectively (Peng et al. 2016). KtrA homologs are more commonly found in LAB than TrkA homologs (Fig. 3). KtrA are noticeably absent in dairy lactococcal species whilst TrkA are only present in streptococci.

The other K^+^ transporter, which has been investigated in LAB is the KUP/HAK/KT family of proteins (IPR003855). Kup homologs are single subunit K^+^/H^+^ importers containing 12 transmembrane spanning domains with an intracellular ∼200 amino acid domain of unknown function. In a salt resistance screen of a high c-di-AMP *gdpP* mutant of *L. lactis*, four single amino acid substitutions in KupB (Kup2) were identified in suppressors (Pham et al. 2018). These mutations were all present on the cytosolic side of the protein, either in the C-terminal domain or in an intracellular loop between TMDs. The K^+^ level, which was reduced in the high c-di-AMP cells compared to wild-type, was increased in cells containing the mutated KupB, indicating gain-of-function changes. In most *L. lactis*, there are two Kup proteins (KupA and KupB), which are encoded by neighbouring genes. Some *L. lactis* strains, including MG1363, have a mutated *kupA* gene that is unable to generate a functional protein (Fig. 3). KupA and KupB from *L. lactis* IL1403 were expressed as full length proteins and shown to bind c-di-AMP using DRaCALA (Quintana et al. 2019). Structural studies revealed that despite having low overall sequence identity, KUP family proteins have conserved structural and key residue consistency with KimA proteins, indicating the latter is a sub-family within the KUP family (Tascon et al. 2020). An attempt to identify the c-di-AMP binding site in the dimeric KimA structure was undertaken, however, no bound nucleotide was identified during cryo-EM analysis (Tascon et al. 2020). The suppressor mutations in KupB that restore salt resistance in a high c-di-AMP mutant of *L. lactis* (Pham et al. 2018) may affect c-di-AMP binding, however, further work is required to verify this and pinpoint the binding pocket.

Two other c-di-AMP receptors involved in potassium homeostasis are the Kdp system and CpaA exporter. KdpDE is a two-component system that regulates expression of the KdpFABC K^+^ importer (Stautz et al. 2021; Fig. 4). The sensor kinase KdpD of *S. aureus* contains an N-terminal USP domain, which binds c-di-AMP (Moscoso et al. 2016). High c-di-AMP in this species resulted in impaired activation of *kdpFABC* transcription upon osmotic stress, which agrees with the inhibitory role c-di-AMP plays on K^+^ accumulation. The K^+^ exporter CpaA is a K^+^/H^+^ antiporter and contains a c-di-AMP binding RCK_C domain (Corrigan et al. 2013). Work in *S. aureus* has found that high c-di-AMP activates K^+^ export by CpaA (Chin et al. 2015), thus lowering intracellular K^+^ levels. Experimental examination of LAB homologs of KdpD or CpaA has not been carried out this far, however, KdpD is present in very few LAB (including *Carnobacterium, L. fermentum* 9–4, and some subspecies of *L. lactis*), while CpaA is also absent from most LAB genera, but common in lactobacilli (Fig. 3).

### Compatible solute transport

#### Glycine-betaine transporter BusA and carnitine transporter OpuC

A number of different compatible solutes are either synthesized or imported by bacteria in response to osmotic stress, including glycine betaine, carnitine, proline, trehalose, and ectoine (Bremer and Kramer 2019). Compatible solutes are preferred over K^+^ as they have less impact on physiological activities within the cell. Most LAB surveyed contain a high affinity ATP-binding cassette (ABC) transporter for glycine betaine termed BusA (OpuA), which consists of two proteins BusAA and BusAB (Fig. 3). The BusA complex consists of an extracellular solute-binding domain (SBD) component, which is commonly fused to a TMD (called BusAB), which interacts with an intracellular ATPase (called BusAA) (Obis et al. 1999, Bouvier et al. 2000). In *L. lactis*, previous work identified that under hyperosmotic conditions, glycine betaine uptake is increased through both higher BusA expression and transporter activity (Obis et al. 1999, Bouvier et al. 2000, van der Heide and Poolman 2000b). The repressor BusR was identified as regulating the transcription of the BusA complex genes by binding to a motif within the promoter region of *busA* in response to ionic strength (Romeo et al. 2003, Romeo et al. 2007). BusA activity is also responsive to ionic strength and membrane composition changes (van der Heide and Poolman 2000a, van der Heide et al. 2001, Biemans-Oldehinkel et al. 2006). A connection between BusA and c-di-AMP was proposed when a *gdpP* mutant of *L. lactis* was found to have five-fold lower *busA* mRNA levels compared to wild type under non-salt-stressed conditions (Smith et al. 2012). Subsequent work in *S. agalactiae* identified inactivating mutations in *busAB*, which permitted growth of a *cdaA* mutant on rich media (Devaux et al. 2018). A *cdaA* mutant of *L. lactis* was also found to harbour a *busA* promoter mutation, which inactivated expression of the glycine betaine transporter (Pham et al. 2021). Together these results suggest that glycine betaine uptake is toxic to low/no c-di-AMP LAB cells. The BusAA ATPase contains a CBS domain, which in other proteins has been found to bind c-di-AMP (Stülke and Krüger 2020). Initial attempts to demonstrate binding between BusAA homologs and c-di-AMP using DRaCALA, however, did not prove successful (Huynh et al. 2016, Devaux et al. 2018).

**Figure 5.**
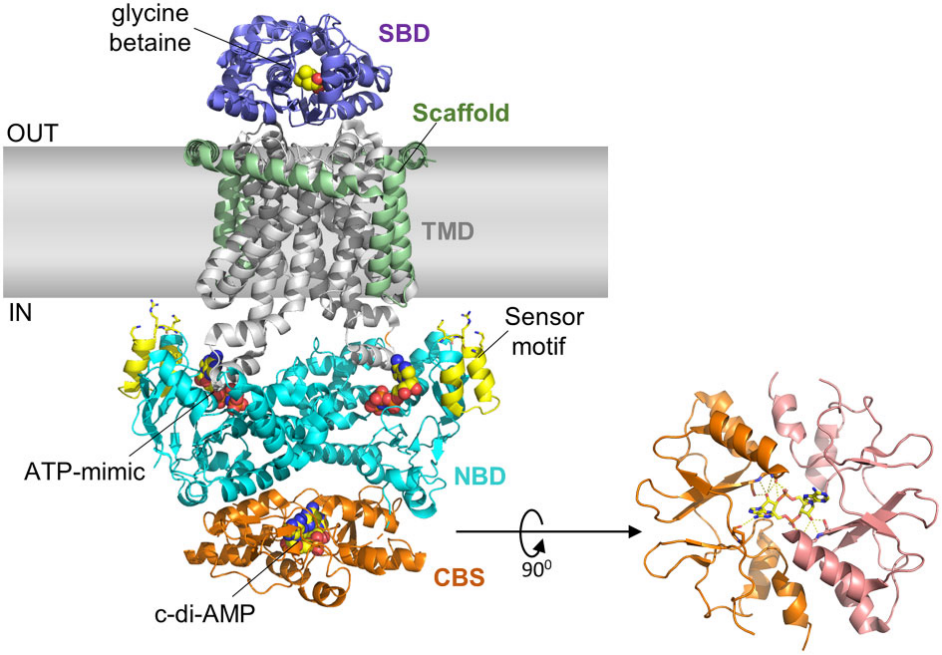
Structure of the *L. lactis* BusA (OpuA) glycine betaine transporter complex obtained by cryo-EM studies. Shown here is the c-di-AMP-inhibited conformation of the ATPase BusAA with c-di-AMP bound at the interface of the tandem CBS domains and the ATP mimic AMP–PNP bound in the NBD domain (Protein Data Bank 7AHH) (Sikkema et al. 2020). In BusAB, glycine betaine bound in the solute-binding protein (SBD), which is fused to the TMD. Highlighted here are also the stabilizing scaffold motif located on the periphery of the TMD of BusAB and the ionic strength sensor motif in BusAA with the functionally important Lys16, Arg17, and Lys19 shown in stick representation.

Recently the cryo-electron microscopy (EM) structural analysis of the BusA transport complex revealed c-di-AMP binding to the CBS domain within the ATPase BusAA component (Sikkema et al. 2020; Fig. 5). This suggests that the DRaCALA method has limitations and that some proteins may need to be in their native membrane complex to exhibit c-di-AMP binding. In addition to confirming the binding of c-di-AMP and glycine betaine by the BusA complex, the cryo-EM study further revealed that BusA is likely to interconvert between different conformations in solution. Based on the structural and function studies, a model was proposed to explain how c-di-AMP regulates the transporter function of BusA. The transport cycle begins with the glycine betaine-bound SBD docking to TMD to form a transient outward-facing conformation. The binding of ATP promotes nucleotide-binding domain (NBD) dimerization to shift BusA into an outward-open conformation to allow glycine betaine to diffuse into a hydrophobic pocket in the TMD. The following ATP hydrolysis induces an inward-facing conformation with the NBDs shifted away from each other. Dissociation of the NBD domains releases glycine betaine to the cytoplasm to complete the transport cycle. Importantly, the release of glycine betaine from BusA is blocked when c-di-AMP binds to the CBS domain to lock BusA into an inhibitory confirmation while stimulating the ATPase activity of the NBD domain. The model agrees with the observations that glycine betaine uptake by membrane reconstituted BusA complex was strongly inhibited by c-di-AMP and the ATPase activity is stimulated by c-di-AMP. The EM studies also implicated a positive charged motif in the NBD domain as a membrane-interacting ionic strength sensor to rationalize the ionic strength dependence of the transporter (Sikkema et al. 2020; Fig. 5).

The ABC transporter for the compatible solute carnitine has also been found to bind c-di-AMP in several LAB (*E. faecalis* and *S. agalactiae*) via the CBS domain in the intracellular ATPase subunit OpuCA (Huynh et al. 2016, Devaux et al. 2018). Using DRaCALA, the *E. faecalis* OpuCA was found to have a K_d_ of 6 µM (Huynh et al. 2016). From our analysis, OpuCA is present in less than half of the LAB examined, including a subset of streptococci and lactobacilli, but absent in lactococci (Fig. 3).

#### Transcriptional repressor BusR

**Figure 6.**
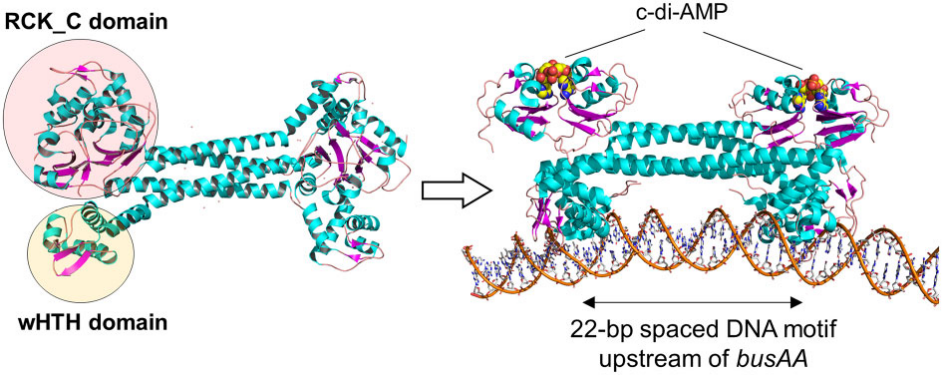
Binding of c-di-AMP promotes BusR-DNA interaction based on the structural studies of *S. agalactiae* BusR. The binding of c-di-AMP to the RCK_C domain of the transcriptional repressor BusR induces a conformational change to release the wHTH domain into a protruding and DNA-interacting position. The c-di-AMP bound BusR subsequently binds to a 22-bp sequence in the promoter region of *busAA* to repress the transcription of the *busAA-AB* genes and hence expression levels of the glycine betaine transporter (Protein Data Bank 7B5Y; Bandera et al. 2021).

*Streptococcus agalactiae* and *L. lactis* BusR bind c-di-AMP via its C-terminal RCK_C domain (IPR006037) and inactivation of *busR* can restore salt resistance to high c-di-AMP *gdpP* mutants (Devaux et al. 2018, Pham et al. 2018). The BusR RCK_C domain binds c-di-AMP with a K_d_ of ∼10 µM (Pham et al. 2018), which is similar to other protein receptors that are mostly in the low micromolar level (He et al. 2020). Glycine betaine levels in *L. lactis* were 10-fold lower in the high c-di-AMP *gdpP* mutant compared to wild-type, consistent with c-di-AMP driving BusR repression (Pham et al. 2018). In agreement with this, glycine betaine levels returned to wild-type levels upon inactivation of *busR*. As previously mentioned, *in vitro* BusR DNA binding is also responsive to ionic strength (Romeo et al. 2007) and high c-di-AMP mutants have reduced K^+^ uptake, so it is likely that c-di-AMP affects BusR repression by direct binding and indirectly by regulating the intracellular ion concentration (Pham et al. 2018). Recent work with *S. agalactiae* BusR has demonstrated that c-di-AMP enhances DNA binding (Bandera et al. 2021). The cryo-EM and crystal structural analysis of *S. agalactiae* BusR showed that the oligomeric BusR adopts an autoinhibited conformation with the DNA-binding winged helix-turn-helix motif (wHTH) domain packed tightly against the RCK_C domain (Fig. 6). Upon the binding of c-di-AMP to the RCK_C domain, BusR shifts to an activated confirmation with the wHTH domain adopting an extended conformation that enables BusR-DNA interaction (Bandera et al. 2021). The DNA-binding specificity is defined by the long coiled-coil region that allow recognition of a 22-bp spaced DNA sequence motif (Fig. 6).

BusR is present in around half of the LAB genomes we analysed and all strains that contained BusR also had BusAA (Fig. 3). A number of species contained BusAA but not BusR, indicating that c-di-AMP regulation of glycine betaine uptake in these bacteria may be solely at the level of inhibiting transporter activity. Interestingly, a number of lactobacilli and streptococci possessed neither the BusR, BusAA, or OpuCA proteins suggesting that there is no c-di-AMP regulation of compatible solute uptake in these species or alternative transporters are present.

### Anionic amino acid biosynthesis

#### Pyruvate carboxylase

Pyruvate carboxylase PycA (PC) catalyses the production of oxaloacetate via the ATP-dependent carboxylation of pyruvate using HCO_3_^−^ (Jitrapakdee et al. 2008). Oxaloacetate is an intermediate metabolite of the tricarboxylic acid (TCA) cycle, however in LAB, the TCA cycle is either incomplete or entirely absent (Morishita and Yajima 1995, Wang et al. 2000a, Willenborg and Goethe 2016). TCA cycle intermediates are instead used in amino acid biosynthesis, including oxaloacetate, which is directly amidated to aspartate by aspartate aminotransferase (AspC; Dudley and Steele 2001). PC is not present in any *Streptococcus, Leuconostoc*, and most lactobacilli analysed. In these LAB, oxaloacetate is instead synthesized from the glycolytic pathway intermediate phosphoenolpyruvate by phosphoenolpyruvate carboxylase (Willenborg and Goethe 2016).

**Figure 7.**
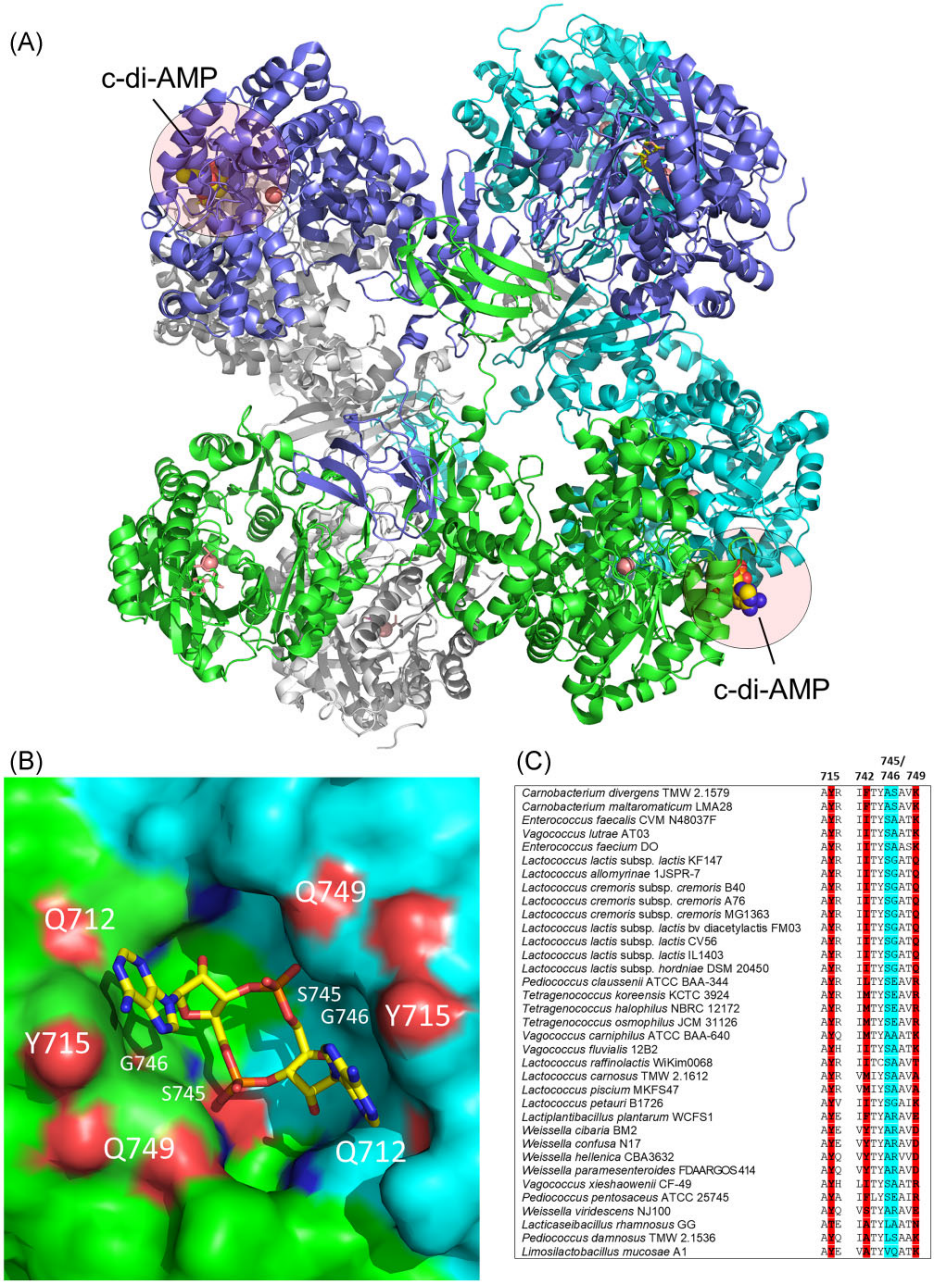
Structure of c-di-AMP bound PC from *L. lactis* (A). The c-di-AMP binding pocket (B) and an alignment of key residues in the c-di-AMP binding pocket of PC in LAB (blue highlighted amino acids should have small side chains in order to not block the c-di-AMP pocket) (C). In the close-up view of the c-di-AMP binding site at the dimeric interface (B), the three key residues (Q712, Y715, and Q749) that are directly involved in c-di-AMP binding are highlighted. The two residues with short side chains (S745, G746) located deeper in the pocket and permit c-di-AMP binding are also highlighted (Protein Data Bank 5VYZ; Choi et al. 2017).

PC was captured in a screen for c-di-AMP receptors in *L. monocytogenes* using a pull-down assay with c-di-AMP coated beads (Sureka et al. 2014). Identification of the c-di-AMP binding pocket from crystal structure analyses and comparison of PC sequences suggested that it is not well conserved and likely that only a subset of PC enzymes can bind this allosteric inhibitor. PC from LAB *E. faecalis* and *L. lactis* were predicted to contain suitable c-di-AMP binding sites and enzyme activity assays revealed up to ∼30% to 60% inhibition by c-di-AMP, respectively (Sureka et al. 2014, Choi et al. 2017). No inhibition by another cyclic dinucleotide c-di-GMP was observed for *L. lactis* PC, demonstrating the specificity of the inhibition (Choi et al. 2017). A crystal structure of the *L. lactis* PC in complex with c-di-AMP was determined to confirm the binding c-di-AMP site (Fig. 7A). *Lactococcus lactis* PC forms a tetramer with c-di-AMP bound at the dimeric interface of two carboxyltransferase domains. A free *L. lactis* PC crystal structure was also determined to show the binding of c-di-AMP induces significant conformational changes and imply that an allosteric mechanism is likely to underly the inhibitory effect of c-di-AMP (Choi et al. 2017). Several residues lining the *L. lactis* PC binding pocket, which are shared with *L. monocytogenes* PC, include a Tyr715 residue that interacts with the adenine base of c-di-AMP and small residues (Ser745 and Gly746) allowing space for c-di-AMP entry into the pocket (Choi et al. 2017) (Fig. 7B). *Staphylococcus aureus* PC, which is not inhibited by c-di-AMP, contains two larger residues (Lys and Gln) at positions 745 and 746 (LlPC numbering) putatively preventing the entry of c-di-AMP (Sureka et al. 2014). We analysed residues from LAB PC that may form a c-di-AMP binding pocket (Fig. 7C). In addition to *Lactococcus* and *Enterococcus*, PC from several other LAB, including *Carnobacterium* and *Vagococcus* may be able to bind c-di-AMP. Other LAB contain one or two residues with longer side chains at positions 745–746, thereby potentially preventing c-di-AMP docking. Therefore, it is likely that only a small subset of LAB PCs are potentially regulated c-di-AMP, however, further work would be required to verify these predictions.

PC and aspartate biosynthesis is essential for acidification of milk by *L. lactis* since nutritional demands for this amino acid are unable to be met by protein/peptide sources (Wang et al. 1998, Dudley and Steele 2001, Choi et al. 2017). Aspartate is a key amino acid in the cell as it is a precursor for five other amino acids as well as pyrimidines (Wang et al. 2000b). In early work with *L. lactis* PC, it was found that >90% of the OA generated by PC is converted to aspartate (Hillier and Jago 1978a). Aspartate was identified as a strong non-competitive inhibitor of *L. lactis* PC activity, with 2.9 mM causing 50% inhibition, (Hillier and Jago 1978b). Unlike other PCs, *L. lactis* PC has high inherent enzyme activity and is insensitive to acetyl-CoA activation (Choi et al. 2017). Together this suggests that the primary role of PC in *L. lactis* is to supply aspartate to the cell.

The finding that c-di-AMP inhibits PC provides an additional layer of regulation of aspartate levels. It was found that a high c-di-AMP *L. lactis* mutant contains lower aspartate levels, which could be restored following introduction of a variant PC (Y715T) that is insensitive to c-di-AMP inhibition (Choi et al. 2017). As well as regulation of biosynthesis in *L. lactis*, aspartate levels are also dampened via indirect c-di-AMP inhibition of import of glutamine via GlnPQ (Pham et al. 2021). Aspartate is the most abundant free amino acid in *L. lactis* and along with glutamate, which is also regulated by c-di-AMP, make up 55% of the free amino acids (Pham et al. 2021). These likely contribute significantly towards the osmotic pressure within the cell and form another extension of the osmoregulatory reach of c-di-AMP via PC activity modulation.

## Stimuli affecting the c-di-AMP pool size

C-di-AMP levels in bacteria change in response to external and internal signals. In agreement with the known targets of c-di-AMP, and the effects this nucleotide plays in osmoregulation, changes that likely affect cellular osmotic pressure have been shown to trigger adjustments in the c-di-AMP level in various LAB. In *S. pneumoniae*, inactivation of the K^+^ transporter gating component and c-di-AMP receptor CabP (KtrA), which likely lowers intracellular K^+^ levels, resulted in a significant reduction of the c-di-AMP level (Zarrella et al. 2018). This work also reported that c-di-AMP levels were lower and CdaA protein levels were reduced in *S. pneumoniae* cells grown in low K^+^ chemically defined media. A role for K^+^ in affecting c-di-AMP levels in *L. lactis* has also been reported with overactive K^+^ uptake (caused by gain-of-function mutations in *kupB* or overexpression of *kupB*) significantly increased c-di-AMP levels (Pham et al. 2018). Also in this study, overaccumulation of the compatible solute glycine betaine, from inactivating *busR*, led to elevated c-di-AMP. Together these findings indicate that intracellular ionic strength and/or turgor changes are sensed by CdaA and/or GdpP to optimize the c-di-AMP concentration. Specifically, low intracellular K^+^ or compatible solute levels, leading to low turgor, trigger the cell to lower the c-di-AMP level in order to activate greater accumulation of osmo-active substances. Conversely, high intracellular K^+^ or compatible solute levels, causing high turgor, trigger the cell to increase the c-di-AMP level in order to inhibit further accumulation of osmo-active substances. As well as these internal signals, external osmolarity changes in the environment have been found to trigger c-di-AMP level adjustments in several bacteria, including the LAB *L. lactis* and *Lb. plantarum* (Pham et al. 2018). Non-growing cells suspended in a low osmolarity solution containing glucose needed for synthesis of the CdaA substrate ATP rapidly increased their intracellular c-di-AMP levels within 5 minutes. The subsequent addition of salt (0.3 M NaCl or KCl) triggered a rapid depletion or block in c-di-AMP synthesis. These studies provide a rational connection between the types of stimuli affecting turgor with the role of many c-di-AMP receptors in osmoregulation. However, the mechanistic basis of how stimuli is transduced into DAC or PDE enzyme activity changes remains to be solved.

Changes in the bacterial cell envelope of *E. faecalis* and *Enterococcus faecium* have also been reported to trigger altered c-di-AMP levels (Wang et al. 2017). Inactivation of *liaR*, which encodes a component of the LiaFSR three-component cell envelope stress response system, led to an elevated c-di-AMP level. Whilst the underlying reason for this is unclear, it is known that the LiaFSR system regulates anionic phospholipid distribution in the cytoplasmic membrane (Tran et al. 2013). This change in membrane composition or charge may affect the activities of CdaA or PDE enzymes, which are both membrane-bound.

In non-LAB, a connection between synthesis of the stringent response signalling molecule (p)ppGpp and c-di-AMP levels through the c-di-AMP binding receptors CbpB (DarB) has been demonstrated (Peterson et al. 2020, Kruger et al. 2021). In turn, (p)ppGpp inhibits the c-di-AMP PDEs GdpP and PgpH (Rao et al. 2010, Huynh et al. 2015), which lead to elevated c-di-AMP levels (Corrigan et al. 2015). Most LAB contain a CbpB homolog (Fig. 3), so it is likely that cross-talk between these two second messengers is also widespread in LAB species.

It is currently not known whether there are stimuli that trigger changes in localized or global c-di-AMP signalling networks. For c-di-GMP, a large number of synthesis and degradation enzymes are present and through the formation of multi-protein complexes with their cognate receptors, cells are able to achieve targeted outputs with a diffusible second messenger (Hengge 2021). Far fewer enzymes are involved in c-di-AMP level homeostasis, however, some LAB have two PDEs. No interactions between c-di-AMP receptors and DAC or PDE enzymes have been reported, but maintaining close proximity to c-di-AMP receptors would allow for faster control of the target receptors activity.

## Extracellular c-di-AMP roles

C-di-AMP released from intracellular and extracellular bacterial pathogens triggers a type I interferon immune response via the stimulator of interferon genes pathway (Woodward et al. 2010, Barker et al. 2013, Andrade et al. 2016). In *S. agalactiae*, extracellular c-di-AMP is hydrolysed by a LPXTG-motif containing cell-wall anchored PDE called CdnP, which results in dampening of the host innate immune response and greater infectivity (Andrade et al. 2016). From our analysis, CdnP is present in only a few LAB (Fig. 3); however, it is unclear if the substrate for this enzyme is c-di-AMP since CdnP shares the same domain structure as another ectonucleotidase NudP, which cleaves AMP to adenosine (Andrade et al. 2016). Interestingly, several of the putative CdnP homologs, including those in *E. faecalis, Vagococcus lutrae*, and *Carnobacterium* have undergone a duplication event and contain two 5′-nucleotidase and metallophosphoesterase domains, resulting in a large cell-wall anchored enzyme.

As observed for bacterial MDR overexpression mutants, elevated extracellular levels of c-di-AMP have also been reported for high c-di-AMP PDE mutants, including *S. pneumoniae* and *L. lactis* (Witte et al. 2013, Pham et al. 2018, Zarrella et al. 2018). Whilst most studies examining the role of c-di-AMP in microbe–host interactions has been in the context of bacterial infections, it is plausible that c-di-AMP released from non-pathogenic food, probiotic, or commensal LAB may also impact host immune responses. Purified c-di-AMP has shown promise as a mucosal adjuvant in vaccine research (Cheng et al. 2022). Work to develop *L. lactis* as a mucosal vaccine delivery system has utilized antigen expressing strains with elevated c-di-AMP, achieved by overexpressing *cdaA* (Quintana et al. 2018). The combination of c-di-AMP and antigen led to a greater immune response than when using only c-di-AMP or antigen expressing strains. Further evidence for a role of c-di-AMP release from non-pathogenic bacteria triggering a host immune response was reported in a recent gut microbiome study (Lam et al. 2021). C-di-AMP released from the common gut commensal bacterium *Akkermansia muciniphila* was identified as a key immune stimulating molecule for effective cancer immunotherapy treatment (Lam et al. 2021).

As well as impacts on the mammalian host, extracellular c-di-AMP has been shown to influence bacterial physiology, specifically rescuing defects of *cdaA* mutants. Exogenously added c-di-AMP to *L. monocytogenes* improved vancomycin resistance (Kaplan Zeevi et al. 2013). In other work, exogenously added c-di-AMP, but not c-di-GMP, was able to restore growth of a *cdaA* mutant of *E. faecalis* in chemically defined media with supplemented peptone, in a dose dependent manner (Kundra et al. 2021). This suggests that extracellular c-di-AMP can potentially be imported by bacteria and go on to regulate intracellular targets. As a result, bacterial cell-to-cell communication via c-di-AMP signalling may potentially occur in high bacterial cell density environments, such as biofilms.

## Future perspectives

- A significant gap in knowledge in the c-di-AMP signalling field is a mechanistic understanding of how external and/or internal signals transduce into c-di-AMP level fluctuations via changes in CdaA and GdpP activity. Studies have reported rapid modulation of c-di-AMP levels upon changing external osmolarity or unbalanced uptake of K^+^ or glycine betaine. This suggests a post-translational (enzyme activity) response. CdaA and/or GdpP may be sensing membrane curvature/stretching alterations or intracellular ionic strength changes via salt-sensitive interactions with membrane phospholipids, similarly to that found for osmosensing compatible solute transporters (Wood 2011, Wood 2015). Ionic strength changes may also potentially affect protein–protein interactions leading to rapid switching to and from catalytically active multimeric enzyme forms. Structural and biochemical studies of full-length and membrane-bound DAC and PDE enzymes may reveal the mechanistic understandings of signal transduction.
- Cells with an imbalanced c-di-AMP level have pleiotropic phenotypes, but are these an indirect result of impaired osmotic homeostasis? Changes in intracellular osmo-active compound concentrations will cause altered turgor pressure that may affect cell envelope properties, such as resistance to cell-wall acting antimicrobials. C-di-AMP controls the levels of dominant intracellular ions, including K^+^ and anionic amino acids (Asp and Glu), which may have global effects in the cell, affecting the activity of enzymes and transporters, as well as macromolecule interactions (e.g. protein–DNA). Teasing apart the reason for various phenotypes is needed to better understand the broad reaching consequences of imbalanced c-di-AMP levels.
- Whilst most LAB contain only one synthesis enzyme and one degradation enzyme, it is less likely that c-di-AMP output specificity requires localized signalling like that observed for more complicated c-di-GMP signalling networks. Several LAB contain two PDE enzymes or only accumulate significant c-di-AMP levels when both DhhP and GdpP are inactivated, suggesting there may be both local and global c-di-AMP signalling networks in some LAB.
- Does extracellular c-di-AMP released from LAB from fermented foods, as probiotics or as part of the microbiome impact on the human host during gastrointestinal transit or colonization? There is evidence of c-di-AMP produced by bacteria in the intestinal lumen that triggers significant host immune responses. As a potent immunostimulatory molecule, future work evaluating c-di-AMP in bacteria–host interactions will be of interest.
- There is the possibility of other as yet unidentified cyclic-dinucleotide systems operating in LAB. A recent study reported the characterization of a cyclic-oligonucleotide-based antiphage signalling system in *L. lactis* involving a 3′,2′-cGAMP-activated nuclease (Fatma et al. 2021). We found this system is absent in our set of representative genomes and therefore unlikely to be a widespread anti-phage system in LAB. Further exploration of alternative nucleotide signalling systems may reveal novel signalling pathways operating in LAB.

## Supplementary Material

fuad025_Supplemental_File
