# Supplementary material for: Cyclic-di-AMP signalling in lactic acid bacteria
Source: FEMS Microbiol Rev. 2023 May 23;47(3):fuad025. doi: 10.1093/femsre/fuad025 (PMC10243994; doi:10.1093/femsre/fuad025)
Supplement: fuad025_Supplemental_File [file fuad025_supplemental_file.docx]

Supplementary Table 1. Phenotypes of c-di-AMP synthesis or degradation gene mutants of LAB.

| **Organism** | **Genotype** | **Phenotype relative to the parent strain*** | **c-di-AMP level** | **References** |
| --- | --- | --- | --- | --- |
| ***Lactococcus lactis*** | Δ*gdpP* | - Resistant to acid (pH=2) killing  - Resistant to high temperature incubation  - Salt sensitive  - Slower milk acidification  - Ampicillin resistant  - Faster cell autolysis  - Thinner cell wall | High c-di-AMP | (Rallu *et al.*, 2000)  (Smith *et al.*, 2012, Zhu *et al.*, 2016, Pham *et al.*, 2021) |
|  | *cdaA* suppressor mutants of Δ*gdpP* | Compared to Δ*gdpP*:  - Salt resistant  - Sometimes salt dependent growth  - Faster autolysis  - Increased cell lysis during growth  - Sometimes cefuroxime sensitive | Low c-di-AMP | (Zhu *et al.*, 2016, Pham *et al.*, 2021) |
| ***Streptococcus***  ***pneumoniae*** | Δ*pde1*^#^ or Δ*pde1*Δ*pde2*^^^ | - Shorter cell chains  - Slower growth  - Sensitive to UV radiation  - Reduced epithelial cell attachment  - Reduced virulence  - Sensitive to heat killing  - Sensitive to slightly acidic (pH=6) growth conditions  - Salt sensitive  - Reduced competence | High c-di-AMP | (Cron *et al.*, 2011, Bai *et al.*, 2013)  (Zarrella *et al.*, 2018)  (Zarrella *et al.*, 2020) |
|  | *cdaA* mutants in Δ*pde1*Δ*pde2* background | Compared to Δ*pde1*Δ*pde2*:  - Faster growth  - Resistant to heat killing  - Resistant to slightly acidic (pH=6) growth conditions  - Improved competence | Low c-di-AMP | (Bai *et al.*, 2013, Zarrella *et al.*, 2018) |
| ***Streptococcus suis*** | Δ*gdpP* | - Slower growth  - Increased biofilm formation  - Reduced hemolytic activity  - Reduced adherence and invasion of Hep-2 cells  - Reduced virulence in mice | High c-di-AMP | (Du *et al.*, 2014) |
| ***Streptococcus mutans*** | Δ*cdaA* | - Reduced biofilm formation^@^  - Reduced colonization of rat oral cavity  - Increased sensitivity to hydrogen peroxide  - Decreased extracellular polysaccharide (EPS) production | No c-di-AMP | (Peng *et al.*, 2016) |
|  | Δ*pdeA*^#^ or Δ*gdpP*Δ*dhhP* | - Slower growth  - Increased EPS production  - Increased biofilm formation  - Increased colonization of *Drosophila* | High c-di-AMP | (Peng *et al.*, 2016, Konno *et al.*, 2018) |
| ***Streptococcus agalactiae*** | Δ*cdaA* | - Unable to grow on rich growth media aerobically  - Unable to grown in chemically defined media with additional glycine-betaine of potassium | No c-di-AMP | (Devaux *et al.*, 2018) |
|  | Δ*gdpP* | - Salt sensitive | High c-di-AMP | (Devaux *et al.*, 2018) |
|  | Δ*cdnP* (ectonuclease) | - Increased IFN-β induction in mice  - Reduced virulence in mice | High extracellular c-di-AMP | (Andrade *et al.*, 2016) |
| ***Streptococcus pyogenes*** | Δ*cdaA* | - Longer lag phase  - Reduced biofilm formation  - Salt sensitive  - Sensitive to slightly acidic (pH=6) growth conditions  - Sensitive to methyl viologen  - Sensitive to ampicillin and vancomycin  - Slower lysis by cell wall hydrolases  - Reduced virulence in mice | No c-di-AMP | (Fahmi *et al.*, 2019, Faozia *et al.*, 2021) |
|  | Δ*gdpP* | - Increased ampicillin resistance  - Reduced virulence in mice  - Faster lysis by cell wall hydrolases | High c-di-AMP | (Cho & Kang, 2013, Fahmi *et al.*, 2019) |
| ***Streptococcus gallolyticus*** | Δ*gdpP* | - Slower growth  - Salt sensitive  - Reduced cell size  - Reduced biofilm formation  - Reduced adherence and cell aggregation on human intestinal cells  - Increased bacteriocin production  - Reduced pilus expression | High c-di-AMP | (Teh *et al.*, 2019) |
| ***Streptococcus thermophilus*** | Δ*cdaA* (Δ*ossG*) | - Sensitive to methyl viologen | N/A | (Thibessard *et al.*, 2004) |
| ***Streptococcus mitis*** | Δ*cdaA* | - Slower growth and reduced final optical density  - Longer cell chains  - Increased cell autoaggregation  - Reduced biofilm formation  - Resistance to ciprofloxacin  - Sensitive to ampicillin or fluoride | No c-di-AMP | (Rørvik *et al.*, 2021)  (Rørvik *et al.*, 2020) |
|  | Δ*pde1* | - Slower growth  - Shorter cell chains  - Sensitive to ciprofloxacin  - Sensitive to UV radiation  - Sensitive to Triton X-100 | High c-di-AMP | (Rørvik *et al.*, 2020, Rørvik *et al.*, 2021) |
| ***Enterococcus faecalis*** | Δ*cdaA* | - Unable to grow on rich growth media, except in the presence of high salt  - Unable to grown in chemically defined media with additional glycine-betaine of carnitine  - Larger cell size  - Increased cell lysis  - Sensitive to bacitracin and daptomycin  - Reduced pilus production  - Reduced biofilm formation  - Reduced survival in serum and urine  - Reduced virulence in *Galleria* *mellonella* and mice | No c-di-AMP | (Kundra *et al.*, 2021) |
|  | Δ*gdpP*Δ*dhhP* | - Sensitive to bacitracin and daptomycin  - Reduced virulence in *Galleria* *mellonella* and mice | High c-di-AMP | (Kundra *et al.*, 2021) |

*Unless indicated, all phenotypes in this table are comparisons between the mutant and wild-type.

^#^Pde1 and PdeA are GdpP orthologs.

^^^Pde2 is a DhhP ortholog.

^@^Different biofilm formation phenotypes have been identified for this species (Cheng *et al.*, 2016)

**References**

Andrade WA, Firon A, Schmidt T, Hornung V, Fitzgerald KA, Kurt-Jones EA, Trieu-Cuot P, Golenbock DT & Kaminski PA (2016) Group B Streptococcus Degrades Cyclic-di-AMP to Modulate STING-Dependent Type I Interferon Production. *Cell Host Microbe* **20**: 49-59.

Bai Y, Yang J, Eisele LE, Underwood AJ, Koestler BJ, Waters CM, Metzger DW & Bai G (2013) Two DHH subfamily 1 proteins in *Streptococcus pneumoniae* possess cyclic di-AMP phosphodiesterase activity and affect bacterial growth and virulence. *Journal of Bacteriology* **195**: 5123-5132.

Cheng X, Zheng X, Zhou X, Zeng J, Ren Z, Xu X, Cheng L, Li M, Li J & Li Y (2016) Regulation of oxidative response and extracellular polysaccharide synthesis by a diadenylate cyclase in *Streptococcus mutans*. *Environ Microbiol* **18**: 904-922.

Cho KH & Kang SO (2013) *Streptococcus pyogenes* c-di-AMP Phosphodiesterase, GdpP, Influences SpeB Processing and Virulence. *PLoS One* **8**: e69425.

Cron LE, Stol K, Burghout P, van Selm S, Simonetti ER, Bootsma HJ & Hermans PW (2011) Two DHH subfamily 1 proteins contribute to pneumococcal virulence and confer protection against pneumococcal disease. *Infect Immun* **79**: 3697-3710.

Devaux L, Sleiman D, Mazzuoli MV, Gominet M, Lanotte P, Trieu-Cuot P, Kaminski PA & Firon A (2018) Cyclic di-AMP regulation of osmotic homeostasis is essential in Group B Streptococcus. *PLoS Genet* **14**: e1007342.

Du B, Ji W, An H, Shi Y, Huang Q, Cheng Y, Fu Q, Wang H, Yan Y & Sun J (2014) Functional analysis of c-di-AMP phosphodiesterase, GdpP, in *Streptococcus suis* serotype 2. *Microbiol Res* **169**: 749-758.

Fahmi T, Faozia S, Port GC & Cho KH (2019) The Second Messenger c-di-AMP Regulates Diverse Cellular Pathways Involved in Stress Response, Biofilm Formation, Cell Wall Homeostasis, SpeB Expression, and Virulence in *Streptococcus pyogenes*. *Infect Immun* **87**.

Faozia S, Fahmi T, Port GC & Cho KH (2021) c-di-AMP-regulated K+ importer KtrAB affects biofilm formation, stress response, and SpeB expression in *Streptococcus pyogenes*. *Infect Immun* **89**.

Konno H, Yoshida Y, Nagano K, Takebe J & Hasegawa Y (2018) Biological and Biochemical Roles of Two Distinct Cyclic Dimeric Adenosine 3',5'-Monophosphate- Associated Phosphodiesterases in *Streptococcus mutans*. *Front Microbiol* **9**: 2347.

Kundra S, Lam LN, Kajfasz JK, Casella LG, Andersen MJ, Abranches J, Flores-Mireles AL & Lemos JA (2021) c-di-AMP Is Essential for the Virulence of *Enterococcus faecalis*. *Infect Immun* **89**: e0036521.

Peng X, Michalek S & Wu H (2016) Effects of diadenylate cyclase deficiency on synthesis of extracellular polysaccharide matrix of *Streptococcus mutans* revisit. *Environ Microbiol* **18**: 3612-3619.

Peng X, Zhang Y, Bai G, Zhou X & Wu H (2016) Cyclic di-AMP mediates biofilm formation. *Mol Microbiol* **99**: 945-959.

Pham HT, Shi W, Xiang Y*, et al.* (2021) Cyclic di-AMP Oversight of Counter-Ion Osmolyte Pools Impacts Intrinsic Cefuroxime Resistance in *Lactococcus lactis*. *mBio* **12**: e00324-00321.

Rallu F, Gruss A, Ehrlich SD & Maguin E (2000) Acid- and multistress-resistant mutants of *Lactococcus lactis* : identification of intracellular stress signals. *Mol Microbiol* **35**: 517-528.

Rørvik GH, Naemi AO, Edvardsen PKT & Simm R (2021) The c‐di‐AMP signaling system influences stress tolerance and biofilm formation of *Streptococcus mitis*. *Microbiologyopen* **10**: e1203-n/a.

Rørvik GH, Liskiewicz KA, Kryuchkov F, Naemi AO, Aasheim HC, Petersen FC, Küntziger TM & Simm R (2020) Cyclic Di-adenosine Monophosphate Regulates Metabolism and Growth in the Oral Commensal *Streptococcus mitis*. *Microorganisms* **8**.

Smith WM, Pham TH, Lei L, Dou J, Soomro AH, Beatson SA, Dykes GA & Turner MS (2012) Heat resistance and salt hypersensitivity in *Lactococcus lactis* due to spontaneous mutation of *llmg_1816* (*gdpP*) induced by high-temperature growth. *Appl Environ Microbiol* **78**: 7753-7759.

Teh WK, Dramsi S, Tolker-Nielsen T, Yang L & Givskov M (2019) Increased Intracellular Cyclic di-AMP Levels Sensitize *Streptococcus gallolyticus* subsp. *gallolyticus* to Osmotic Stress and Reduce Biofilm Formation and Adherence on Intestinal Cells. *J Bacteriol* **201**.

Thibessard A, Borges F, Fernandez A, Gintz B, Decaris B & Leblond-Bourget N (2004) Identification of *Streptococcus thermophilus* CNRZ368 genes involved in defense against superoxide stress. *Appl Environ Microbiol* **70**: 2220-2229.

Zarrella TM, Metzger DW & Bai G (2018) Stress suppressor screening leads to detecting regulation of cyclic di-AMP homeostasis by a Trk-family effector protein in *Streptococcus pneumoniae*. *J Bacteriol* **200**.

Zarrella TM, Yang J, Metzger DW & Bai G (2020) Bacterial Second Messenger Cyclic di-AMP Modulates the Competence State in Streptococcus pneumoniae. *J Bacteriol* **202**: 1.

Zhu Y, Pham TH, Nhiep THN*, et al.* (2016) Cyclic-di-AMP synthesis by the diadenylate cyclase CdaA is modulated by the peptidoglycan biosynthesis enzyme GlmM in *Lactococcus lactis.* *Mol Micro* **99**: 1015–1027.
